# The cerebral mechanism of the specific and nonspecific effects of acupuncture based on knee osteoarthritis: study protocol for a randomized controlled trial

**DOI:** 10.1186/s13063-020-04518-5

**Published:** 2020-06-23

**Authors:** Na Zhang, Jin-Ling Li, Chao-Qun Yan, Xu Wang, Lu-Lu Lin, Jian-Feng Tu, You-Sheng Qi, Jun-Hong Liu, Cun-Zhi Liu, Li-Qiong Wang

**Affiliations:** 1 School of Acupuncture-Moxibustion and Tuina, Shandong University of Chinese Medicine, Jinan, Shandong China; 2 Acupuncture Research Center, School of Acupuncture-Moxibustion and Tuina, Beijing University of Chinese Medicine, No. 11, Bei San Huan Dong Lu, Chaoyang District, Beijing, 100029 China; 3 School of Life Sciences, Beijing University of Chinese Medicine, Beijing, China; 4 Nanyuan Community Health Service Center, Fengtai District, Beijing, China

**Keywords:** Specific effect, Nonspecific effect, Acupuncture, Knee osteoarthritis, Functional magnetic resonance imaging, Clinical trial

## Abstract

**Background:**

Research on the effect of acupuncture has been limited. Whether the effect of acupuncture is equivalent to placebo has been the focus of debate in this field. This study will explore the specific and non-specific effects of acupuncture for knee osteoarthritis (KOA) by functional magnetic resonance imaging (fMRI).

**Methods and design:**

Ninety participants diagnosed with KOA will be randomly divided into the acupuncture group, sham acupuncture group, and waiting list group in a ratio of 1:1:1. Except for the waiting list group, the other participants will receive acupuncture or sham acupuncture three sessions per week for 4 weeks respectively. The primary outcome will be the response rate which is defined on an individual basis as at least a 2-point decrease in the numerical rating scale (NRS) of pain at the end of intervention period compared with the baseline. fMRI scans will be performed at baseline and the end of the intervention period to examine the response of various brain regions. The secondary outcomes will include the Western Ontario and McMaster Osteoarthritis Index (WOMAC), State-Trait Anxiety Scale-State Anxiety Subscale (STAI-S), and Stanford Expectations of Treatment Scale (SETS). Pearson’s correlation coefficient will be performed to investigate the changes in brain activity and clinical variables.

**Discussion:**

The results of our study will help to evaluate the specific and nonspecific effects of acupuncture combined with clinical and brain function changes based on KOA.

**Trial registration:**

Chinese Clinical Trial Registry ChiCTR1900025799. Registered on 9 September 2019.

## Background

The therapeutic effect of the intervention may be the result of its specific effect and nonspecific effect [1]. In this progress, the nonspecific effect of treatment (e.g., acupuncture) may be caused by accidental intervention factors, such as expectation and emotion [2]. The term “placebo effect” is thought to partially explain the nonspecific effects of these treatments [3]. The cumulative evidence suggested that the placebo effect was a real psychobiological event that was attributable to the interaction between the participant, clinician, and treatment environment in the overall treatment context [4–6]. However, the clinical impact of the placebo is largely overlooked, especially that the placebo effect in a randomized controlled trial (RCT) is usually subtracted from the treatment effect [7].

In recent years, accumulating evidences from RCTs suggest that acupuncture was effective compared with no treatment [8]. At the same time, some large randomized clinical trials comparing acupuncture with sham or placebo acupuncture found that the therapeutic effect of acupuncture is greatly reduced, indicating that the nonspecific effect (placebo effect) of acupuncture may play a part role [9–11]. A randomized controlled trial attributed the acupuncture effect to a powerful placebo effect [12]. Although previous studies supported that the holistic effect of acupuncture included specific and nonspecific effects [13, 14], but the proportion of the effects and their determinants were still unclear. Understanding comprehensively the effect of acupuncture and integrating the nonspecific effects into clinical practice (for example, improving treatment expectations) may help to optimize the holistic effect [15]. Therefore, in the selection and implementation of interventions, it becomes increasingly important that nonspecific be separated from specific effects. In order to evaluate the importance of nonspecific effects, the sham acupuncture was used as the control to further distinguish the acupuncture effect in this study design.

Along with the use of neuroimaging techniques in pain research, there are growing evidences that the neural markers of the brain can predict pain perception and response to drugs or non-drug therapy [16–18]. Since neuroimaging may provide objective markers of the effects of treatments on the organism, it enables a more nuanced and biology-based interpretation of RCT outcomes, where active treatment and inert placebo treatment might be differentiated at the brain circuit level [19]. Pascal et al. showed that clinical placebo response had biological underpinnings by using functional connectivity to predict placebo responders and non-responders. The differences in the whole-brain map identified four brain regions that distinguished placebo responders from non-responders; the right medial frontal gyrus (r-MFG) was the most significant, followed by posterior cingulate cortex (PCC), anterior cingulate cortex (ACC), and the right secondary somatosensory and primary motor cortex (r-s2/M1) [20]. Another RCT study examined the effects of placebo drugs on brain activity in participants with chronic back pain; it found that the placebo analgesia depended on the structure and function of the brain [21]. In recent years, functional magnetic resonance imaging (fMRI) has been used to investigate the central regulatory mechanism of acupuncture effect [22, 23]. Previous neuroimaging studies showed that acupuncture could regulate the functional connections of pain-related networks [24–28]. During analgesia, acupuncture could restore the critical brain areas to change the attention and memory associated with pain, especially the periaqueductal gray (PAG), medial frontal cortex (MFC), and bilateral hippocampus (Hpc) [29]. However, the brain activity regulated by placebo acupuncture is usually neglected, which leads to insufficient evidence to identify the neurological mechanisms triggered by the specific and nonspecific effects of acupuncture.

The pain was the most common research topic in acupuncture-related studies published from 1994 to 2014, accounting for about one third of the total publications [30]. A cross-sectional study found that among the common acupuncture adaptation diseases, 5 of the top 10 diseases were related to pain [31]. Knee osteoarthritis (KOA) is high-prevalence chronic osteoarthritis with knee pain and functional limitation [32–34]. The positive response of placebo treatment is a recognized phenomenon in the chronic pain, and the analgesic effect is often comparable to active treatment [35]. For these reasons, KOA is an appropriate model to study the specific and nonspecific effects of acupuncture.

The purposes of this study are to (1) investigate the cerebral mechanism of the specific (true acupuncture vs sham acupuncture) and nonspecific effects (sham acupuncture vs waiting list) of acupuncture based on the brain activities by using fMRI and (2) investigate the proportion of specific and nonspecific effects in acupuncture treatment.

## Methods and design

### Study design

**Fig. 1:**
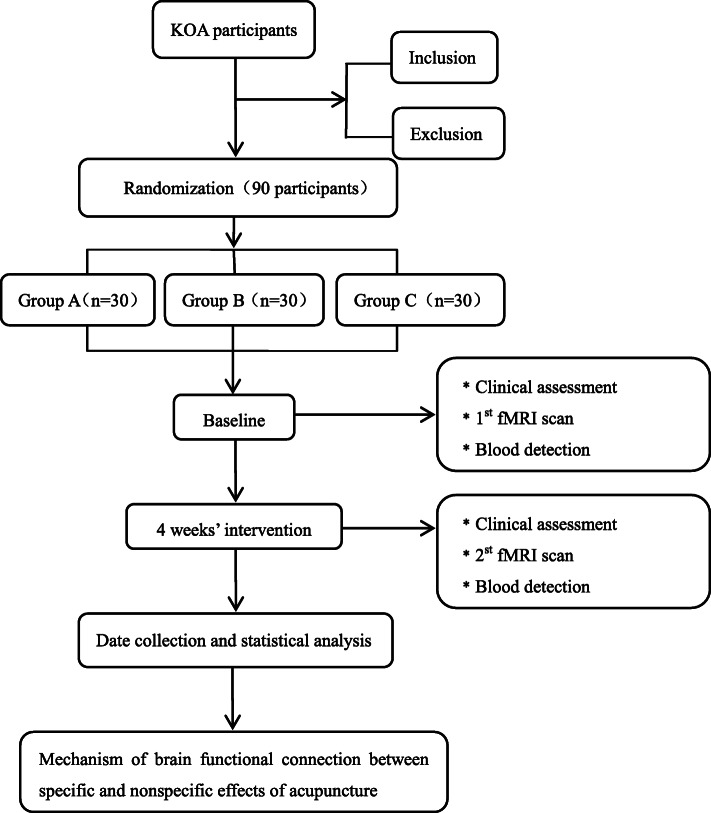
Flowchart of the study design. KOA, knee osteoarthritis

**Fig. 2:**
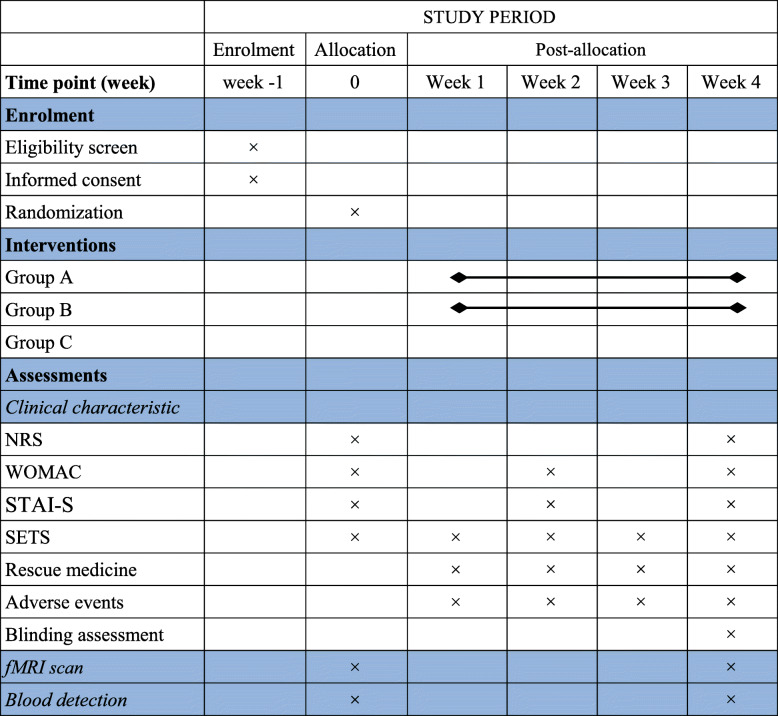
Schedule of enrollment, intervention, and assessment. NRS, Numerical rating scale; WOMAC, Western Ontario and McMaster Universities Osteoarthritis Index; STAI-S, State-Trait Anxiety Scale-State Anxiety Subscale; SETS, Stanford Expectations of Treatment Scale; fMRI, functional magnetic resonance imaging

This is a randomized controlled, parallel study to explore whether specific and nonspecific effects of acupuncture regulate certain brain functional connectivity, respectively. Ninety participants will be recruited in the study, with an average of 30 in each group. Clinical data measurements and fMRI scans will be evaluated at baseline and the end of treatment (Figs. 1 and 2). The study has been approved by the ethical committees of Dongzhimen Hospital Affiliated to Beijing University of Chinese Medicine (NO: DZMEC-KY-2017-53-02) and registered in the Chinese Clinical Trial Registry (NO: ChiCTR1900025799). The protocol will be reported following Standard Protocol Items: Recommendations for Interventional Trials (SPIRIT) statement (Additional file 1).

### Study setting and recruitment

The study will be carried out at the Dongzhimen Hospital Affiliated to Beijing University of Chinese Medicine. The study will recruit participants with KOA who meet the diagnostic criteria of the American College of Rheumatology criteria [36] through hospital outpatient, WeChat official account (one of china’s popular social media platforms) of Dongzhimen Hospital and brochures. Meanwhile, the recruitment of participants has started in October 2019.

### Participants

#### Inclusion criteria

(1) Age between 45 and 65 years old, and gender is not limited; (2) knee pain lasts at least 6 months; (3) KL (Kellgren-Lawrence) grade II or III in recent 12 months; (4) numerical rating scale (NRS) ≥ 4 in the past week; (5) right handedness; and (6) signed informed consent.

#### Exclusion criteria

(1) History of knee replacement or waiting surgery (knee replacement or knee arthroscopy); (2) history of arthroscopy within 1 year or intra-articular injection within 6 months; (3) discomfort of knee joint caused by other reasons (such as joint cavity effusion, infection, malignant tumor, autoimmune disease, trauma, etc.); (4) serious organic pathological changes, mental abnormalities, and psychiatric or neurological disorders; (5) coagulation disorders; (6) pregnant or lactating women; (7) MRI contraindications, such as claustrophobia, implanted with pacemaker and other metal products; (8) severe skull anatomical asymmetry or definite lesions were found in magnetic resonance scanning; (9) received acupuncture or massage treatment in recent 1 month; (10) allergy to needles and alcohol or fear to acupuncture; and (11) participated in other clinical studies in the past 3 months.

### Informed consent and participant safety

Participants will be required to sign an informed consent (Additional file 2) and have the same chance to receive appropriate treatment. During the treatment, any adverse events, such as panic, subcutaneous hematoma, and pain, should be informed to the study researchers, who are responsible for monitoring and recording the adverse events and reactions throughout the study. If the participants cannot tolerate these adverse reactions, acupuncture treatment will be discontinued and the participants will be withdrawn from the study. There is no anticipated harm and compensation for study participation.

### Randomization

In this study, KOA participants will be randomly divided into acupuncture group, sham acupuncture group, and waiting list group in a ratio of 1:1:1, with 30 participants in each group. The random sequence will be generated by an independent professional statistician (LQ. Wang) who is not involved in the study with the software SAS 9.3 (SAS Institute, Cary, NC, USA) using block randomization. The random numbers will be stored by a fixed non-involved person. When the eligible participants are randomly assigned, the random number will be told by the fixed person to the acupuncturists via a short message.

### Blinding

Due to the operation characteristics of acupuncture, the acupuncturists cannot be blinded who will be informed of the grouping of participants before treatment. Participants will be told that they will randomly receive one of the interventions after enrollment. To avoid communication, participants will be assigned to different rooms during the treatment. Since the participants assigned to waiting treatment do not have any intervention, it is impossible to blind them. The outcome evaluators and data statisticians will be blinded to the group allocation, and their responsibility is to collect and analyze the data.

### Sample size

According to previous neuroimaging studies, in order to calculate statistical power for group-level fMRI studies, a sample size of 12 participants were required to achieve 80% power at the single voxel level when *α* = 0.05, and about 25 participants were required for a stricter threshold of *α* = 0.000002 [37, 38]. In this study, considering a 20% dropout rate and loss of data due to head motion, we plan to recruit 30 participants in each group. Each participant will receive 2 fMRI scans to explore different brain activity between the specific and nonspecific effects of acupuncture.

### Interventions

#### Group A—acupuncture treatment

**Fig. 3:**
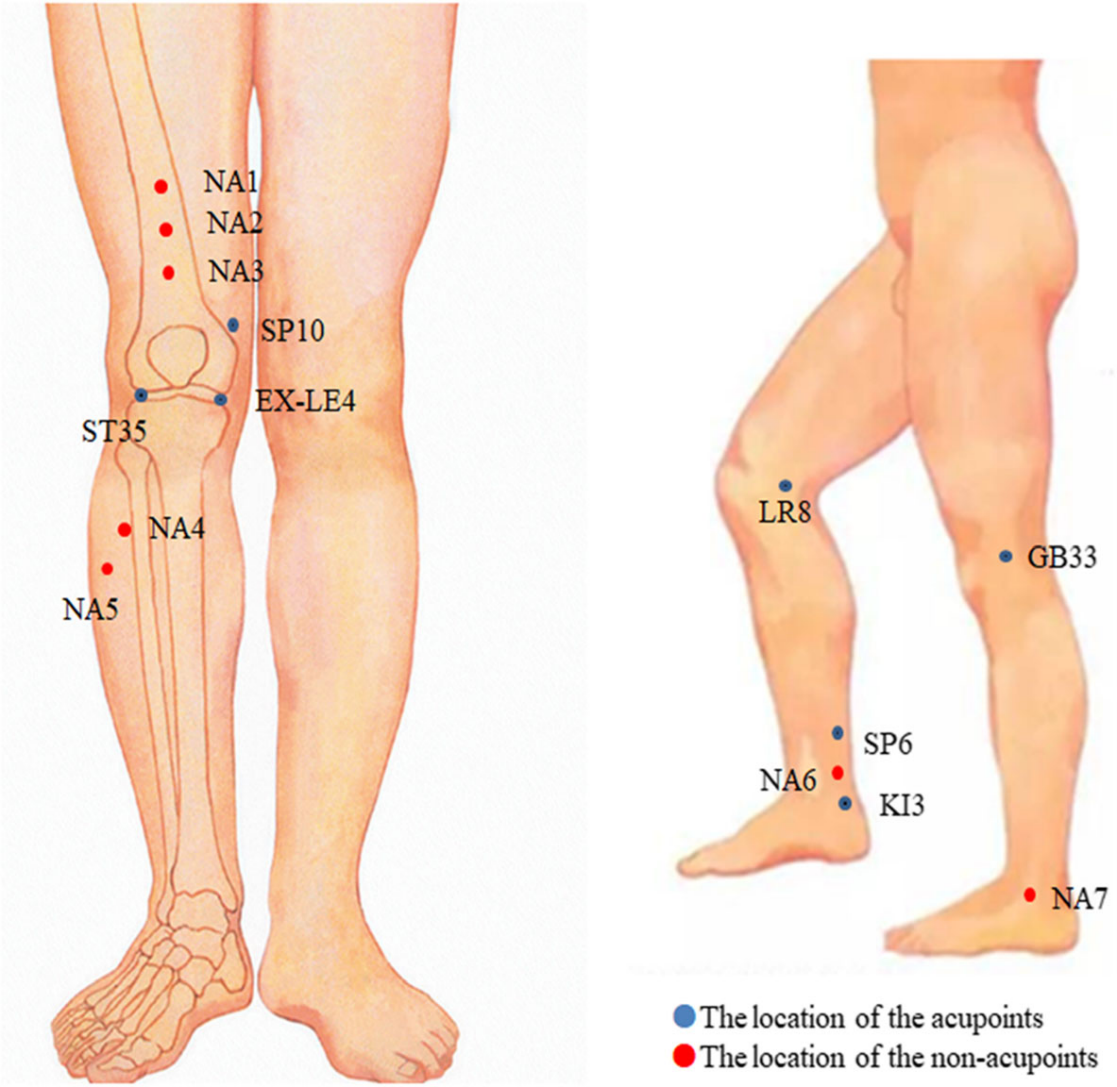
The location of acupoints. Red circles: location of non-acupoints. Blue circles: location of acupoints. Abbreviation: ST35, Dubi; EX-LE4, Neixiyan; LR8, Ququan; GB33, Xiyangguan; SP10, Xuehai; SP6, Sanyinjiao; KI3, Taixi; NA, non-acupoint

**Fig. 4:**
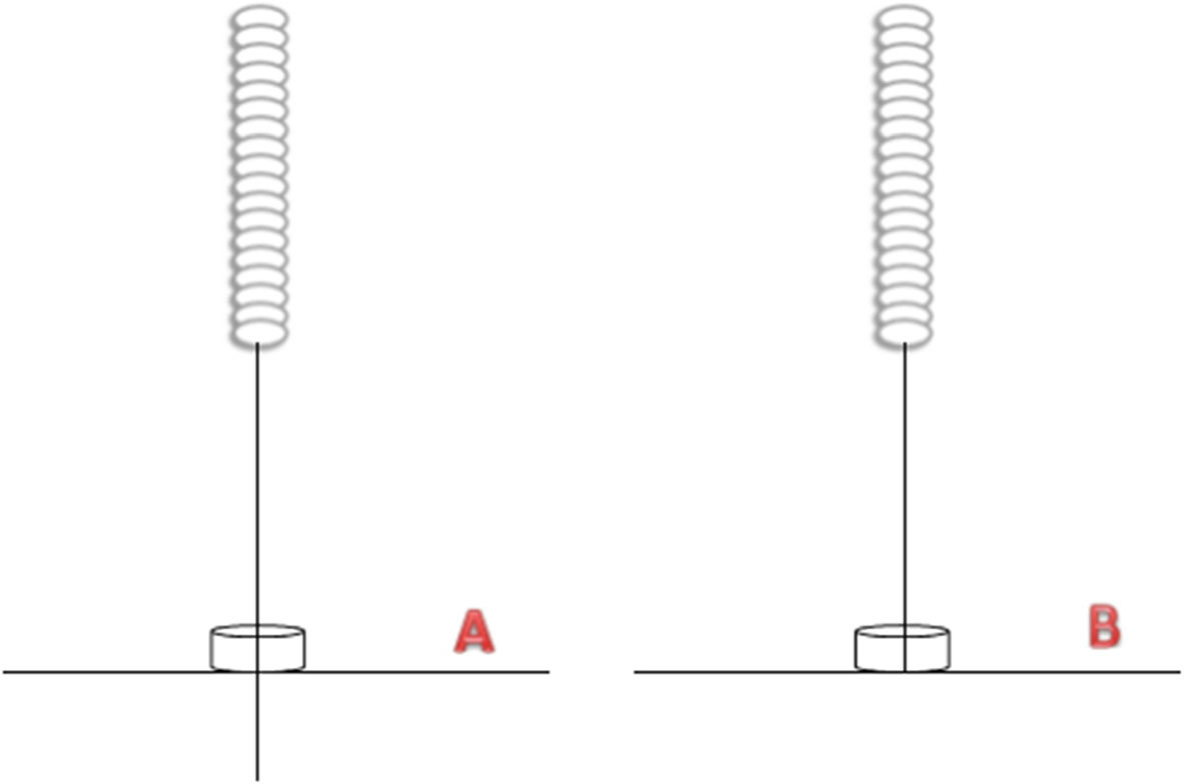
The picture of acupuncture and sham acupuncture

Participants in group A will receive 12 sessions of manual acupuncture treatment (3 sessions weekly for 4 weeks). The acupoints will include dubi (*ST35*), neixiyan (*EX-LE4*), ququan (*LR8*), xiyangguan (*GB33*), xuehai (*SP10*), sanyinjiao (*SP6*), taixi (*KI3*), and an ashi point (the point where the patient feels most pain) (Table 1 and Fig. 3). First, the adhesive pad will be applied on the skin of the acupoints according to WHO Standard Acupuncture Locations, and then single-use acupuncture needles (0.25 * 40 mm or 0.25 * 25 mm, Hwato, Suzhou, China) will be inserted into acupoints through the adhesive pad (Fig. 4). Acupuncturists will manually stimulate the needles to achieve de qi. The needles will be left for 30 min.

**Table 1:** Locations of acupoints for acupuncture

Acupoints	Locations^a^
Dubi (ST 35)	On the anterior aspect of the knee, in the depression lateral to the patellar ligament
Neixiyan (EX-LE4)	On the anterior aspect of the knee, in the depression medial to the patellar ligament
Ququan (LR8)	On the medial aspect of the knee, in the depression medial to the tendons of the semitendinosus and the semimembranosus muscles, at the medial end of the popliteal crease
Xiyangguan (GB33)	On the lateral aspect of the knee, in the depression between the biceps femoris tendon and the iliotibial band, posterior and proximal to the lateral epicondyle of the femur
Xuehai (SP10)	On the anteromedial aspect of the thigh, on the bulge of the vastus medialis muscle, 2 cun superior to the medial end of the base of the patella
Sanyinjiao (SP6)	On the tibial aspect of the leg, posterior to the medial border of the tibia, 3 cun superior to the prominence of the medial malleolus
Taixi (KI3)	On the posteromedial aspect of the ankle, in the depression between the prominence of the medial malleolus and the calcaneal tendon

^a^1 cun (≈ 20 mm) is defined as the width of the interphalangeal joint of the participant’s thumb

#### Group B—sham acupuncture

Participants in group B will receive non-insertive acupuncture treatment on non-acupoints using the sham needles with blunt tip (0.25 × 40 mm or 0.25 × 25 mm, Hwato, Suzhou, China) (Fig. 4). Similar to the acupuncture group, the adhesive pad will be applied to the non-acupoints. Sham needles will not penetrate the skin and will not require the “De Qi” sensation. The needles will be left for 30 min. The acupuncture treatment will include 12 sessions for 4 weeks (3 sessions per week). The location of the non-acupoints is shown in Table 2 and Fig. 3.

**Table 2:** Locations of non-acupoints for sham acupuncture

Acupoints	Locations^a^
NA1	On the anterior aspect of the thigh, 6 cun above the upper edge of the patella (between the spleen and stomach meridian)
NA2	On the anterior aspect of the thigh, 5 cun above the upper edge of the patella (between the spleen and stomach meridian)
NA3	On the anterior aspect of the thigh, 4 cun above the upper edge of the patella (between the spleen and stomach meridian)
NA4	In the middle of GB34 and ST36 (between the gallbladder and bladder meridian)
NA5	3 cun below GB34 (between the gallbladder and bladder meridian)
NA6	2 cun above the medial malleolus (between the liver and spleen meridian)
NA7	In the middle of GB40 and ST41 (between the gallbladder and bladder meridian)

*NA* non-acupoints

^a^1 cun (≈ 20 mm) is defined as the width of the interphalangeal joint of the participant’s thumb

#### Group C—waiting list

Participants in group C will not receive acupuncture within 4 weeks. After 4 weeks of observation, participants will receive 12 sessions of acupuncture treatment as free compensation.

During the treatment period, some other interventions, such as moxibustion, cupping, and massage, will not be allowed. Participants will withdraw from the study if they violate the protocol. When their pain is unbearable, they will be allowed to take non-steroidal anti-inflammatory drugs. Researchers should keep relevant records.

### Clinical outcome assessments

The primary outcome will be the response rate, which is defined as a decrease of more than 2 points on the NRS at week 4 compared with baseline [39]. NRS is a line divided into 10 segments. The degree of pain is assessed by 0–10 scores, and 0 score represents pain-free and 10 score represents the worst pain.

The secondary outcomes will be as following: (1) Pain subscale of Western Ontario and McMaster Osteoarthritis Index (WOMAC) scale will be used to evaluate the degree of knee pain of the participants. (2) Function subscale of the WOMAC scale will be used to evaluate the knee joint function. (3) Stiffness subscale of WOMAC will be used to evaluate the stiffness of knee joint. The severity of knee joint will increase with the higher scores of WOMAC. Participants will fill out the WOMAC scale at weeks 0, 2, and 4. (4) Blinding assessment and rescue medicine. (5) Stanford Expectations of Treatment Scale (SETS) will be used to assess participants’ positive and negative expectations of the upcoming treatment and their overall understanding of the purpose of the treatment. Participants will be asked to fill out the SETS scale before weekly treatment. (6) State-Trait Anxiety Scale-State Anxiety Subscale (STAI-S) will be used to evaluate the severity of current anxiety symptoms in participants with knee osteoarthritis. Participants will fill out the STAI-S scale at weeks 0, 2, and 4.

### MRI data acquisition

The MRI scan will be performed with a 3.0 Tesla superconductor (Skyra, Siemens, Erlangen, Germany) in the Beijing Hospital of Traditional Chinese Medicine Affiliated to Capital Medical University. Participants will undergo fMRI scans at baseline and the end of intervention. Before scanning, participants will be required to wear earplugs and headgear and remove all magnetic and metal items. The foam pad will be used to reduce head movement. Participants will be required to maintain a comfortable supine position and keep awake.

Scanning parameters: echo planar imaging (EPI) sequence will be used for resting fMRI scanning: repetition time (TR) = 2000 ms, echo time (TE) = 30 ms, field of view (FOV) = 224 mm × 224 mm, flip angle (FA) = 90°, layer thickness = 3.5 mm, axial slices = 32, size of voxel = 3.5 mm × 3.5 mm × 3.5 mm, in-plane resolution = 64 × 64, volumes = 240.

### Neurotransmitter and genetics assessment

Current neurophysiological studies showed that genetic variations in the neurotransmitter pathway mediating the placebo effect increased the possibility of identifying placebo responders through gene screening [40]. Given the subjectivity of clinical scales measurement, oxytocin, catechol-o-methyltransferase (COMT), and opioid receptor (OPRM1) will be tested to assess and predict the specific and nonspecific effects of acupuncture.

Participants will be fasted for at least 8 h before collecting the blood at the baseline, and venous blood will be drawn from the upper arm in the morning. Blood samples will be immediately centrifuged into two parts, plasma and blood cells, and will be stored in a refrigerator at − 80 °C. Finally, the samples will be processed in a technical laboratory by standard methods.

### Data management and monitoring

The case report form (CRF) will be used to record major clinical data, adverse events, and safety assessments during the study, with a unique numeric identifier for each participant. The clinical research assistant will validate the accuracy, missing, and consistent data in CRF. Clinical questionnaires will be administered to all participants in a separate room and administered by the same research. The clinical data will be independently entered into the EpiData electronic database by two researchers and tested by referring to the original data source when inconsistent data appears. EpiData electronic data will be exported to Microsoft Excel and then analyzed using SPSS software package (SPSS 12.0 ko for Windows). The participants will be scanned by a professional imaging doctor on the same machine, and after each scan, professional technicians will check the quality of the imaging data.

### Statistical analysis

#### Clinical data analysis

The clinical data will be statistically analyzed by SPSS software (SPSS V.22.0 KO for Windows). Data will be described utilizing the mean (standard deviation) when following a normality assumption and using the median (quartile spacing) when the normality assumption is violated. The percentage (*n* %) will be used to describe the categorical variables. Comparisons between the three groups will be made by repeated-measures analysis of variance or Wilcoxon rank-sum (if normality is violated). For the primary outcome, we will calculate response rates at 4 weeks using the chi-square test. For the secondary outcomes, the mixed effect model and repeated measurement method will be used to compare the continuous variables (including WOMAC, SETS, and STAI-S).

The primary outcome will be performed using the intention-to-treat (ITT) and per-protocol (PP) analysis. ITT is defined as all randomized participants completing at least one post-baseline measurement. PP is defined as all the randomized participants who will not stop treatment prematurely, and complete 4 weeks of treatment, and undergo two MRI scans. The last observation carried forward (LOCF) will be used to estimate the missing values. The data will be considered statistically significant with a level of 0.05, and all hypothesis tests will be two-tailed.

#### Neuroimaging data analysis

Imaging data will be analyzed using Data Processing Assistant and Resting-State fMRI (DPARSF) toolbox performing on MATLAB 8.6 (Mathworks, Inc., Natick, MA, USA). Data preprocessing will include DICOM format conversion, eliminating the first 10 time points, time correction, head motion correction, spatial standardization (re-sampled to 3 mm × 3 mm × 3 mm), removing linear trend, low-frequency filtering (0.01–0.1 Hz), and regression removal of covariates (head model parameters, cerebrospinal fluid, brain white matter, and the mean whole-brain signals).

After data preprocessing, the amplitude of low-frequency fluctuation, regional homogeneity, functional connectivity analysis, or voxel-wise degree centrality will be performed to investigate the brain responses of different groups. *T* test and repeated-measures analysis of variance will be conducted to investigate the differences in brain regions in each group. Pearson correlation analysis will be used to test the correlation between fMRI imaging data and clinical variables.

### Ethics and dissemination

The protocol was designed following the principles of the Helsinki declaration. Participants will be informed of the study protocol, possible risks, and other related matters before entering the study and sign the informed consent before randomization. The results will be published in peer-reviewed academic journals and disseminated through conferences.

## Discussion

To evaluate the specific and nonspecific effects of acupuncture, we designed a randomized, controlled, three-arm clinical trial. Although some studies explained the specific effect of acupuncture, the effect of acupuncture is equivalent to the placebo effect that has been the focus of debate in the field. As far as we know, our study will be the first parallel, randomized study of specific and nonspecific effects of acupuncture in combination with clinical questionnaire evaluations such as patient expectations, and functional magnetic resonance imaging. In this study, different acupuncture interventions will be conducted in participants with KOA to observe the correlation between clinical manifestations and changes in brain activity, so as to further understand and identify the specific and nonspecific effects of acupuncture.

In recent years, acupuncture, as a non-drug treatment, has been widely recognized and studied in the world [41]. However, with the application of acupuncture in the worldwide, more and more high-level research teams and magazines pay attention to the effect of acupuncture in clinical research [42–45]. Although some studies raised doubts and challenges on the specific effect of acupuncture, it cannot be denied that the nonspecific effect of acupuncture is a key part of clinical effect [46]. Besides, few studies compared the proportion of specific and nonspecific effects of acupuncture in the holistic effect [13, 14]. To sum up, optimizing the nonspecific effect of acupuncture to improve the overall efficacy of acupuncture is a problem worthy of attention. Therefore, a better understanding of the neural mechanisms underlying the specific and nonspecific effects of acupuncture is crucial to achieving these goals. Based on this, our study will explore the related neural mechanism of the effect of acupuncture using fMRI to explore the full potential of acupuncture.

In order to avoid the bias of results and improve the reliability of clinical results, we try to keep the baseline consistency as much as possible. Participants will be screened strictly according to inclusion and exclusion criteria. At the same time, We will also evaluate the influence of participants’ expectations and anxiety on the intervention to observe the state of participants’ emotions on the effect of acupuncture and brain activities. All the researchers will be trained to understand the design of the study. To achieve blindness in the course of the intervention, the adhesive pad should be attached to acupoints and non-acupoints before the intervention. In order to maintain the consistency of acupuncture techniques, acupuncturists have at least 5 years of experience. For fMRI, all of participants will be carried out in the fMRI room of Chinese Medicine affiliated to Capital Medical University.

This study also has several limitations. First of all, acupuncturists and participants in the waiting list will not be blinded due to the nature of the intervention. Secondly, the long-term efficacy of acupuncture will not be observed due to the observation treatment period of the study is only 4 weeks. Finally, our study may ultimately provide only a possible explanation for the small sample size included in each group.

### Trial status

The study protocol was approved by the Ethical Committee of Dongzhimen Hospital Affiliated to Beijing University of Chinese Medicine on 29 August 2019 (version 2.0, 25 June 2019). The study is currently in the recruitment phase, and the first participant was included in 30 October 2019. We predict that recruitment will be completed by December 2021.

## Supplementary information

**Additional file 1.** SPIRIT 2013 Checklist.

**Additional file 2.** Model consent form.

## Data Availability

The full protocol will be available from the corresponding author after identification. Data sets generated or analyzed during the study will not be made public until they are published in a peer-reviewed international journal.

## Abbreviations

NRS: Numerical rating scale
WOMAC: Western Ontario and McMaster Universities Osteoarthritis Index
STAI-S: State-Trait Anxiety Scale-State Anxiety Subscale
SETS: Stanford Expectations of Treatment Scale
fMRI: Functional Magnetic Resonance Imaging
NA: Non-acupoint
KOA: Knee osteoarthritis
CRF: Case report form
ITT: Intention-to-treat analysis
PP: Per-protocol
LOCF: Last observation carried forward
