# Supplementary material for: The cerebral mechanism of the specific and nonspecific effects of acupuncture based on knee osteoarthritis: study protocol for a randomized controlled trial
Source: Trials. 2020 Jun 23;21:566. doi: 10.1186/s13063-020-04518-5 (PMC7310547; doi:10.1186/s13063-020-04518-5)
Supplement: Supplementary file 2 — Additional file 2. Model consent form. [file 13063_2020_4518_MOESM2_ESM.doc]

**患者知情同意书告知页（1）**

**(版本号：3.0、版本日期：2019.08.08)**

我们将要开展一项“针刺治疗膝骨关节炎的随机对照研究II”，您的情况可能符合该项研究的入组条件，因此，我们想邀请您参加该项研究。本知情同意书将向您介绍该研究的目的、步骤、获益、风险、不便或不适等，请仔细阅读后慎重做出是否参加研究的决定。当研究者向您说明和讨论知情同意书时，您可以随时提问并让他/她向您解释您不明白的地方。您可以与家人、朋友以及您的医生讨论之后再做决定。

若您目前正参加其他临床研究，请告知您的研究医生或者研究人员。

本项研究的项目负责人是北京中医药大学东直门医院王军教授，本项研究受北京市科委重大项目资助 。

**为什么进行这项研究？**

临床上安慰剂效应普遍存在，针刺相关的临床试验带来的不一致性结论很大程度上是对针刺特异性效应及非特异性效应机制缺乏明确共识。针刺起效的关键是否等价于针刺安慰效应一直是领域内争论的焦点。为了明确针刺特异性与非特异性的临床效应机制，结合静息态功能磁共振技术，以**膝骨关节炎（knee osteoarthritis, KOA）患者**为研究对象，明确针刺特异性与非特异性效应的存在及响应脑区。

**哪些人将被邀请参加这项研究？**

本项研究将邀请符合以下纳入排除标准（以下任意一项为“否”不能参加试验。）：

（1）年龄45-65岁之间，男女不限；

（2）膝关节疼痛，病程超过6个月；

（3）12个月以内的放射检查显示KL分级为Ⅱ或Ⅲ级；

（4）过去一周内疼痛数字评分法（NRS）≥4；

（5）右利手；

（6）签署知情同意书者。

排除标准（以下任意一项为“是”不能参加试验。）：

（1）有膝关节手术史或正在等待膝关节手术（膝关节置换或膝关节镜手术）；

（2）评价关节1年之内有关节镜检查史、近6个月有关节腔注射史；

（3）其他疾病引起的膝部疼痛（如关节游离体、关节腔严重积液、感染、恶性肿瘤、自身免疫疾病、外伤、骨折、痛风等）；

（4）严重的急性或慢性器质性或精神类疾病；

（5）凝血功能障碍疾病（如血友病等）；

（6）备孕、妊娠期及哺乳期妇女；

（7）幽闭恐惧症者或体内携带心脏起搏器、金属制品等其他MRI检查禁忌者；

（8）MRI扫描中发现有明确器质性病变或严重头部解剖结构不对称者；

（9）近1月内接受推拿或针灸治疗；

（10）对针具和酒精过敏或者恐惧扎针者；

（11）近3个月内参加其他临床研究者。

**多少人将参与这项研究？**

本研究计划招募90名**膝骨关节炎**患者。

**该研究是怎样进行的？**

本研究将历时4周左右。如果您自愿参加本项研究，我们希望您配合进行一下事宜：

1. 纳入研究前，您的研究医生将参照纳入排除标准，询问您的相关情况，并告知您研究的相关情况，解答所有有关的疑问；
2. 如果您进入试验，您将会有相同的机会进入针刺治疗组、新型安慰针刺治疗组、等待治疗组，分组概率为1:1:1，即您进入其中一组的概率为1/3，并接受相应的治疗，治疗方式如下：
3. 三组选穴数目相同，数量都为7个穴位，单侧膝痛针单侧膝关节，双侧膝痛针双侧膝关节；
4. 针刺组和等待治疗组选穴相同，穴位为：曲泉、犊鼻、内膝眼、膝阳关、血海、三阴交、太溪；新型针刺组在非传统经脉上选取七个穴点为固定穴位；
5. 针刺组和等待治疗组针刺方法为传统针刺方法，以得气为度；新型针刺组采用特制针具（0.25*40mm,华佗牌）对非穴点进行刺激，不得气。

3、您将于研究开始前完成疼痛数字评分量表、西安大略和麦克马斯特大学骨关节炎指数评分量表、特质-焦虑量表、斯坦福期望治疗量表等评价针刺对患者疼痛和功能的影响；在针刺治疗第1周±3天、治疗第2周±3天、治疗第3周±3天、治疗第4周±3天完成疼痛数字评分量表、斯坦福期望治疗量表；在针刺治疗第2周±3天、治疗第4周±3天完成西安大略和麦克马斯特大学骨关节炎指数评分量表、状态-特质焦虑分量表；在第4周±3天时完成功能磁共振扫描和血液采集，血液检测需每次抽取受试者血液10ml，用于检测催产素、多巴胺和血清素，儿茶酚-O-甲基转移酶（Comt）、阿片受体（Oprm1）、脂肪酸酰胺水解酶（Faah）和催产素受体基因（Oxtr），我们也将通过针刺不良反应评价观察针刺治疗的安全性。

4、整个试验期间，您可在疼痛时向医生领取对乙酰氨基酚/西乐葆（200mg/次，1次/天）或乐松（60mg/次，3次/天）作为临时口服止痛药物，并在日记卡上详细记录服药情况。请您保管好所有的包装及剩余的药物，试验结束后您的医生将这些材料回收留档。

**参加该研究对受试者日常生活的影响？**

当您决定是否参加本研究时，请仔细考虑如上所列的检查和随访对您的日常工作、家庭生活等可能的影响。考虑每次回访的时间与交通问题。若您对试验涉及的检查和步骤有任何疑问，可以向我们咨询。

研究期间需要禁止服用非本试验提供的药物。

考虑到您的安全以及为确保研究结果的有效性，在研究期间您不能再参加其他任何有关药物和医疗器械的临床研究。

**参加本研究受试者的风险和不良反应？**

若试验期间，您发生任何不良反应或不适，您应立刻向研究医生报告，这是至关重要的。如果您或您的研究医生认为您无法耐受这些不良反应，针灸治疗可能会完全停用，您可能会退出本研究。

**针刺的风险**

针刺过程中随时进行不良反应评价包括血肿、晕针、针刺后遗感等情况。

**影像的风险**

在扫描过程中极少数不自知患有幽闭恐惧症的患者会感到恶心、胸闷、心慌等症状。

**参加本研究受试者可能的获益？**

研究表明，针刺可以镇痛，改善膝骨关节炎相关症状。本研究应用每周3次的针刺治疗膝骨关节炎，可能能够改善膝痛、功能障碍等症状，提高患者生活质量。同时我们希望从您参与的这项研究中得到的信息在将来能够使与您病情相同的病人获益。

**如果不参加此研究，有没有其他备选治疗方案？**

您可以选择不参加本项研究，这对您获得常规治疗不会带来任何不良影响。目前针对您的健康情况，常规的治疗方法有：口服非甾体类抗炎药、止痛药、葡萄糖氨基酸、仙灵骨葆胶囊等中成药或汤药、局部外用药物、关节腔注射或关节镜手术，严重者可选择关节置换。

**是否一定要参加并完成本项研究？**

您是否参加这个研究完全是**自愿的**。如果您不愿意，可以拒绝参加，这对您目前或未来的卫生医疗不会有任何负面影响。即使您同意参加之后，您也可以在任何时间改变主意，告诉研究者退出研究，您不会因退出试验而遭到歧视或报复，也不会影响您获得正常的医疗服务。当您决定不再参加本研究时，希望您及时告知您的研究医生，研究医生可就您的健康状况提供建议和指导。

根据方案中止/退出标准，告知受试者出现以下情况会中止其继续参加研究。（1）出现严重不良事件者，根据医生判断应该停止该病例临床试验者。（2）出现其他影响试验观察的病证，根据医生判断应该停止临床试验者，作无效病例处理。（3）临床试验方案实施中发生了重要偏差，如依从性太差等，难以评价针灸疗效。（4）受试者在临床试验过程中不愿意继续进行临床试验，向主管医生提出退出临床试验的要求者。

监管机构也可能在研究期间终止本研究。如果发生本研究提前终止的情况，我们将及时通知您，您的研究医生会根据您的健康状况为您下一步的治疗计划提供建议。

对于中途退出的受试者，出于安全性考虑，我们有末次随访计划，您有权拒绝。若您退出后，发现新的与您健康和权益相关的信息时，我们可能会再次与您联系。

受试者退出后，需明确今后将不收集与其有关的新数据。并对如何处理之前收集的研究数据及因不良反应退出的数据向受试者做出细致说明。

**参加该项研究的费用**

### 本研究的12次针刺治疗、对乙酰氨基酚/西乐葆或乐松、2次功能磁共振与血液的检查均由研究单位承担。

本研究无报酬、交通费、误工费等补偿。

**发生研究相关伤害的处理？**

当您的健康状况在参加本研究期间受到伤害时，请告知研究者（张娜，联系电话18615639141），我们会采取必要的医疗措施，如治疗期间病情加重，可增加药物治疗。根据我国相关法规条例规定，发生研究相关的伤害时，本项研究的课题组将承担相应的医疗费用及对此提供相应的经济补偿。

**若参加研究，我需要做什么？**

- 提供准确的既往病史和当前病情信息。
- 告诉研究医生您在研究期间出现的任何健康问题。
- 告诉研究医生您在研究期间服用的任何新药、药物、维生素或草药。
- 除非经过研究医生许可，否则不应服用任何药物或治疗，包括处方药和在药店柜台购买的药品（包括维生素和草药）。
- 按医嘱接受针刺治疗，按要求访视。
- 记录日志卡，并在每次访视时携带日志卡。
- 不要参加其它医学研究。
- 遵循研究人员和研究医生的指导。
- 有任何不清楚的地方您可以随时询问。

**受试者的个人信息会得以保密吗？**

如果您决定参加本项研究，您参加研究及在研究中的个人资料均属保密。所有的研究成员都被要求对您的身份保密。您的档案将保存在有锁的档案柜中，仅供研究人员查阅。为确保研究按照规定进行，必要时，政府管理部门或伦理委员会的成员按规定可以在研究单位查阅您的个人资料。这项研究结果发表时，将不会披露您个人的任何资料。

**与研究相关的新信息？**

在试验过程中我们可能会获知有关治疗的新信息，我们会及时通知您，让您决定是否继续参加研究或退出。

**研究结束之后是否继续提供研究针刺治疗？**

研究结束后，课题组将不再继续向您提供针刺治疗。

**如果有问题或困难，该与谁联系？**

如果您有与本研究相关的任何问题，请联系张娜，联系电话18615639141。

如果您有与受试者自身权益相关的问题，可与北京中医药大学东直门医院医学伦理委员会联系，联系电话：010-010-84012709。

**第二部分 知情同意签名页**

**受试者知情同意声明**

我已被告知基于**膝骨关节炎**探讨针刺特异性和非特异性效应的脑功能连接机制研究项目的研究的背景、目的、步骤、风险及获益情况。我有足够的时间和机会进行提问，问题的答复我很满意。我也被告知，当我有问题，或想进一步获得信息，应当与谁联系。我已经阅读这份知情同意书，并且同意参加本研究。我知道在研究期间任何时刻无需任何理由我都可以退出本研究。我被告知我将得到这份知情同意书的副本，上面包含我和研究者的签名。

受试者签名： 日期：

联系电话：

法定代理人签字【如适用】： 日期：

与受试者关系：

我确认，在知情同意书中的信息是被正确解释了的并且受试者和/或受试者合法代表明白理解了这些信息。受试者自愿同意参加本研究。

公平见证人签名【如适用】： 日期：

**研究者告知声明**

我已告知该受试者（和其法定代理人）基于**膝骨关节炎**探讨针刺特异性和非特异性效应的脑功能连接机制研究项目的研究背景、目的、步骤、风险及获益情况，给予他/她足够的时间阅读知情同意书、与他人讨论，并解答了其有关研究的问题；我已告知该受试者当遇到问题时的联系方式；我已告知该受试者（或法定代理人）他/她可以在研究期间的任何时候无需任何理由退出本研究。

研究者签名： 日期：

联系电话：
